# Long-term outcome and predictors of long-term disease activity in natalizumab-treated patients with multiple sclerosis: real life data from the Austrian MS Treatment Registry

**DOI:** 10.1007/s00415-021-10559-w

**Published:** 2021-04-22

**Authors:** Michael Guger, Christian Enzinger, Fritz Leutmezer, Franziska Di Pauli, Jörg Kraus, Stefan Kalcher, Erich Kvas, Thomas Berger, the Austrian MS Treatment Registry (AMSTR)

**Affiliations:** 1 Clinic for Neurology 2, Med Campus III, Kepler University Hospital GmbH, Krankenhausstr. 9, 4021 Linz, Austria; 2 Medical Faculty, Johannes Kepler University Linz, Linz, Austria; 3 Department of Neurology, Medical University of Graz, Graz, Austria; 4 Department of Neurology, Medical University of Vienna, Vienna, Austria; 5 Clinical Department of Neurology, Medical University of Innsbruck, Innsbruck, Austria; 6 Department of Laboratory Medicine, Paracelsus Medical University and Salzburger Landeskliniken, Salzburg, Austria; 7 Department of Neurology, Medical Faculty, Heinrich-Heine-University, Düsseldorf, Germany; 8 Hermesoft, Data management, Graz, Austria; 9 Hermesoft, Statistics, Graz, Austria

**Keywords:** Disability progression, Long-term, Multiple sclerosis, Natalizumab, Predictor

## Abstract

**Objectives:**

To evaluate long-term effectiveness of natalizumab (NTZ) and to determine demographic, clinical, and radiological predictors regarding long-term disease activity (≥ 7 years) in a nationwide observational cohort, using data collected prospectively in a real-life setting.

**Materials and methods:**

We analysed data from 230 patients from the Austrian Multiple Sclerosis Treatment Registry (AMSTR), who had started treatment with NTZ at any time since 2006 and stayed on NTZ for at least 7 years without treatment gap of more than three months.

**Results:**

Estimated mean annualised relapse rates (ARR) over a mean treatment period of 9.3 years were 0.07 for NTZ. Sustained EDSS progression for 12 weeks was observed in 36 (19%) patients and for 24 weeks in 31 (16.3%) cases. Sustained EDSS regression for 12 and 24 weeks was seen in 45 (23.7%) and 42 (22.1%) cases. The baseline parameters ≥ 1 Gadolinium-enhancing MRI lesion(s) [incidence rate ratio (IRR) of 0.409 (95% CI 0.283–0.593), *p* = 0.001], ARR ≤ 1 in the prior 12 month before treatment initiation with NTZ [IRR of 0.353 (95% CI 0.200–0.623), *p* = 0.001] and EDSS ≤ 1 [incidence rate ratio (IRR) of 0.081 (95% CI 0.011–0.581), *p* = 0.012] were significantly associated with a reduced relapse risk, whereas a disease duration ≤ 5 years increased significantly the ARR [IRR of 1.851 (95% CI 1.249–2.743), *p* = 0.002]. The only predictive baseline parameter for experiencing EDSS progression (sustained for 12 and 24 weeks) was age > 35 years [HR of 2.482 (95% CI 1.110–5.549), *p* = 0.027, and HR of 2.492 (95% CI 1.039–5.978), *p* = 0.041, respectively].

**Conclusions:**

These real-life data show a stable disease course regarding relapse activity and disease progression under NTZ treatment for more than 7 years. The main predictors for disease activity were higher relapse rate before treatment initiation, higher disability, shorter disease duration and absence of Gadolinium-enhancing MRI lesions at baseline. Older age at NTZ start was the only significant risk factor for disease progression over long-term.

## Introduction

High treatment efficacy of natalizumab (NTZ) for relapsing remitting multiple sclerosis (RRMS) has been proven in various trials [1–5]. In comparison to placebo, NTZ reduced the annualised relapse rate (ARR) by 68% and sustained progression of disability by 42% [1].

However, the use of NTZ may be associated with John-Cunningham virus (JCV) induced progressive multifocal leukoencephalopathy (PML). Risk factors for PML are anti-JCV antibody positivity, prior use of immunosuppressive drugs and duration of NTZ treatment, in particular if longer than 2 years. Therefore, NTZ treatment discontinuation is considered after risk stratification in patients with a high likelihood of PML [5, 6].

However, little is known about predictors for the long-term outcome of RRMS patients treated with NTZ. In previous studies, longer disease duration, older age, lower pre-treatment relapse activity and higher Expanded Disability Status Scale (EDSS) scores at NTZ initiation have been defined as unfavourable predictors for disease progression [7–9], NTZ discontinuation due to lack of efficacy [10, 11] and experiencing a relapse [12]; whereas, higher ARR at baseline, lower baseline EDSS, younger age and shorter disease duration were favourable predictors for no evidence of disease progression [7] and disease improvement [12–14]. In contrast, a lower baseline ARR showed a higher chance maintaining no further inflammatory disease activity in other studies [3, 12, 15]. Nevertheless, the observation periods were so far limited. These discrepancies and limitations prompted us to further investigate this issue to confirm or rebut published findings.

The objectives of our study were first to evaluate long-term effectiveness of NTZ and, second, to determine demographic, clinical and radiological predictors for long-term disease activity (≥ 7 years) including time to first relapse, ARR, EDSS progression and regression in a nationwide observational cohort using data collected prospectively in a real-life setting.

## Materials and methods

### Data collection

The Austrian MS Treatment Registry (AMSTR) [16–18], established in 2006 to maintain quality control and comply with reimbursement regulations of the Austrian sick funds, allows to obtain clinical data, to assess indications, the clinical profiles of the treated patients and to monitor safety in real life. The AMSTR is part of the dense network of MS centres in Austria, which is constituted by MS clinics from neurological departments and some dedicated neurological practices that have been assigned this status by the Austrian Society of Neurology based on defined quality criteria. In addition, prescriptions of disease modifying therapies (DMTs) for MS are exclusively reserved for MS centres. Thus, prescriptions and treatment documentations are evenly distributed across Austria. The AMSTR is compliant with Austrian laws on bioethics and it was also approved by the ethical committee of the Medical University of Vienna (EC number 2096/2013).

AMSTR documents anonymous baseline data, including the date of clinical onset of MS and disease duration, relapses in the prior 12 months, EDSS, magnetic resonance imaging (MRI) activity and previous DMT. Follow-up data (relapses, EDSS, adverse events [AEs], change or discontinuation of treatment) are required to be documented every 3–6 months. Each relapse has to be confirmed by a neurologist at the MS centre and documented in the AMSTR. Documentation also requires date of relapse onset, EDSS and use/dose of i.v. methylprednisolone treatment. Besides the fact that applying the AMSTR is mandatory for reimbursement, a special quality-related feature of the AMSTR is an external and independent data monitoring to improve data acquisition, input and management in terms of completeness and plausibility of documented data.

In 2006, the European Medicines Agency (EMA) approved NTZ and reimbursement for NTZ in Austria adheres to this approval. Thus, NTZ-treated patients in Austria either have to have had at least one relapse in the prior 12 months despite treatment with interferon beta or glatiramer-acetate and at least nine T2 lesions or at least one Gadolinium (Gd)-enhancing lesion on recent brain MRI (“indication A”), or two or more severe relapses in the preceding treatment-naïve 12 months and one or more Gd-enhancing lesions on brain MRI or a significant increase in T2 lesion load as compared to a previous recent MRI (“indication B”).

We investigated a cohort of 230 patients (long-term cohort) from the AMSTR, who had started treatment with NTZ at any time since 2006 and continued NTZ treatment for at least 7 years without treatment gap of more than three months.

The primary outcome measure was the ARR under treatment with NTZ during this period. Relapses were defined as new or worsening neurological symptoms lasting for at least 24 h in the absence of fever.

Further outcome measures were time to first relapse and sustained disability progression or regression. Sustained disability progression or regression was defined as an increase or decrease from baseline of at least 1.0 point in the EDSS score (or at least 0.5 points for patients with a baseline EDSS score greater than 5.5) that persisted for at least 12 or 24 weeks.

Based on these outcome measures we analysed baseline parameters (age, gender, disease duration, indication, pre-treatment ARR, EDSS and MRI [number of T2 lesions, number of Gd-enhancing T1 lesions]) as possible predictors for the further disease course.

For analyses of treatment interruption of NTZ, we defined two causes, i.e., (a) permanent treatment interruption and (b) treatment interruption and re-start with any new medication.

### Statistical methods

All models included the following factors derived from the patient’s baseline values: age over 35 years, sex, presence of at least 9 MRI T2 hyperintense lesions and at least one Gd-enhancing MRI T1 lesion, ARR greater than 1, duration of disease over 5 years, EDSS greater than 1.0, and previous disease-modifying treatment.

We used a generalised linear model (GLM) with relapse count as Poisson distributed dependent variable and treatment period as offset variable to find factors influencing ARR.

Cox proportional hazards models were used to search for factors-affecting time to first relapse, EDSS progression confirmed after 12 and 24 weeks, and EDSS regression confirmed after 12 and 24 weeks.

Identification of thresholds for dichotomising predictive baseline factors started with the models described above and multiple levels of the baseline factors (e.g. levels for relapse count 12 months before baseline: 0–1, 2, 3, > 3). In the next step, the neighbouring levels with the clearest change in relative event probability (IRR, HR) were identified. If such a position could not be identified uniquely, clinical considerations were considered. All levels below and above this threshold were combined generating the dichotomised factor (e.g. levels of baseline ARR: 0–1, > 1).

We used IBM SPSS Statistics for Windows, Version 24.0 (Armonk, NY: IBM Corp.) as statistical software.

## Results

According to the defined inclusion criteria, 230 (long-term cohort) out of 1665 (total cohort) NTZ-treated RRMS patients could be included in this study for further analyses. From the 230 patients, we had to exclude further 40 cases for determining predictors because of missing baseline values, especially MRI data. Demographic and descriptive clinical data are shown in Table 1.

**Table 1:** Baseline patient characteristics

	NTZ ≥ 7 years *N* = 190 11.4%	NTZ ≥ 7 years^a^ *N* = 230 13.8%	NTZ < 7 years *N* = 1435 86.2%	Total *N* = 1665 100%
Female				
* N*	142	163	1020	1183
%	74.7%	70.9%	71.1%	71.1%
Age^b^				
Mean	35.7	35.9	34.8	35
SD	9	9	10.2	10
Duration of MS at baseline (years)^b^				
Mean	8.2	8.0	7.2	7.3
SD	5.8	6	6.1	6.1
EDSS at baseline^b^				
Mean	3.1	3.2	2.8	2.9
SD	1.5	1.5	1.7	1.7
Relapse rate within 12 months prior treatment start^b^				
Mean	2.3	2.3	2.1	2.1
SD	1.2	1.2	1.1	1.1
≥ 9 T2 lesions				
Yes				
*N*	179	212	1320	1532
%	94.2%	95.1%	93.7%	93.9%
No				
*N*	11	11	89	100
%	5.8%	4.9%	6.3%	6.1%
≥ 1 Gd-enhancing T1 lesion				
Yes				
*N*	129	133	932	1065
%	67.9%	68.6%	69.2%	69.2%
No				
*N*	61	61	414	475
%	32.1%	31.4%	30.8%	30.8%
Indication^c^				
A				
*N*	147	180	1021	1201
%	77.4%	79.3%	73.6%	74.4%
B				
*N*	41	47	367	414
%	21.8%	20.7%	26.4%	25.6%
Follow-up in years				
Mean	9.2	9.3	3.0	3.9
SD	1.5	1.5	2.2	3

*EDSS* Expanded Disability Status Scale, *Gd* gadolinium, *MS* multiple sclerosis, *NTZ* natalizumab, *SD* standard deviation

^a^Missing baseline parameters included

^b^Comparison between cohort NTZ ≥ 7 years and cohort < 7 years NTZ using Mann–Whitney *U* test revealed p value < 0.05

^c^Indication A = at least 1 relapse in the prior 12 months despite treatment with either interferon beta or glatiramer-acetate; indication B = at least two severe relapses in the prior 12 months in treatment-naive patients

**Fig. 1:**
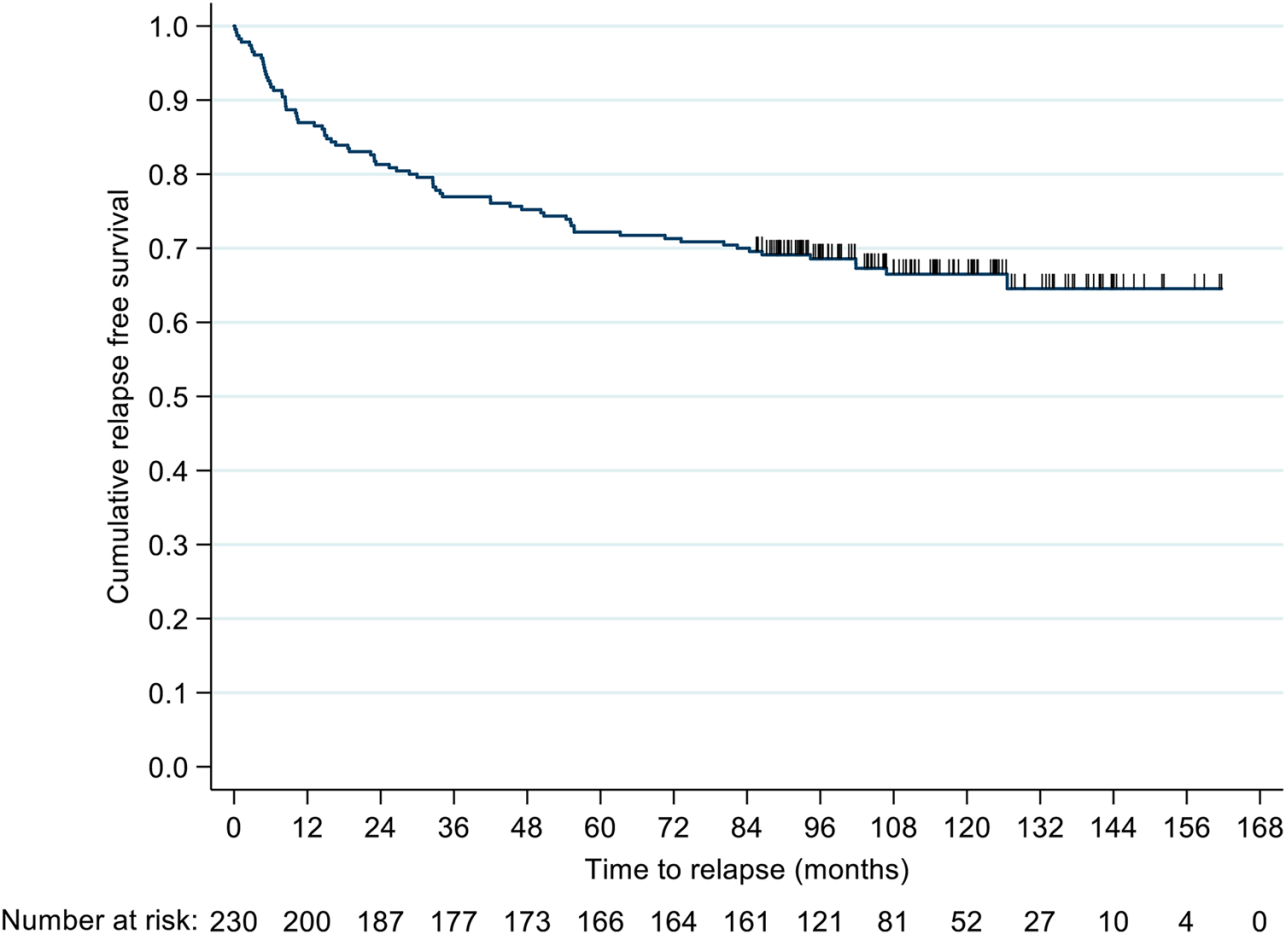
Cumulative survival without relapse during natalizumab treatment in the long-term cohort within the observation period

Mean treatment periods for the 230 RRMS patients were 9.3 (SD 1.5) years. 76 (33%) patients treated with NTZ experienced a relapse during the observation period, accounting for a mean annualised relapse rate (ARR) of 0.07 (SD 0.15) (Fig. 1).

In contrast, the cohort < 7 years of NTZ treatment (short-term cohort, n = 1435) showed a mean ARR of 0.22 (SD 0.58) over a mean treatment period of 3.0 (SD 2.2) years (*p* = 0.268).

The mean EDSS change in the long-term group was − 0.08 (= improvement; SD 1.5), with a mean EDSS of 3.2 at baseline and 3.2 at the last follow-up visit.

**Fig. 2:**
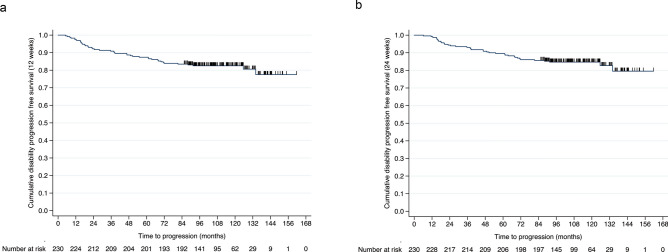
**a**, **b** Cumulative survival without disease progression sustained for 12 **a** and 24 **b** weeks during natalizumab treatment in the long-term cohort within the observation period

Sustained EDSS progression confirmed at week 12 and week 24 was observed in 36 (19%) and in 31 (16.3%) patients (Fig. 2a, b).

**Fig. 3:**
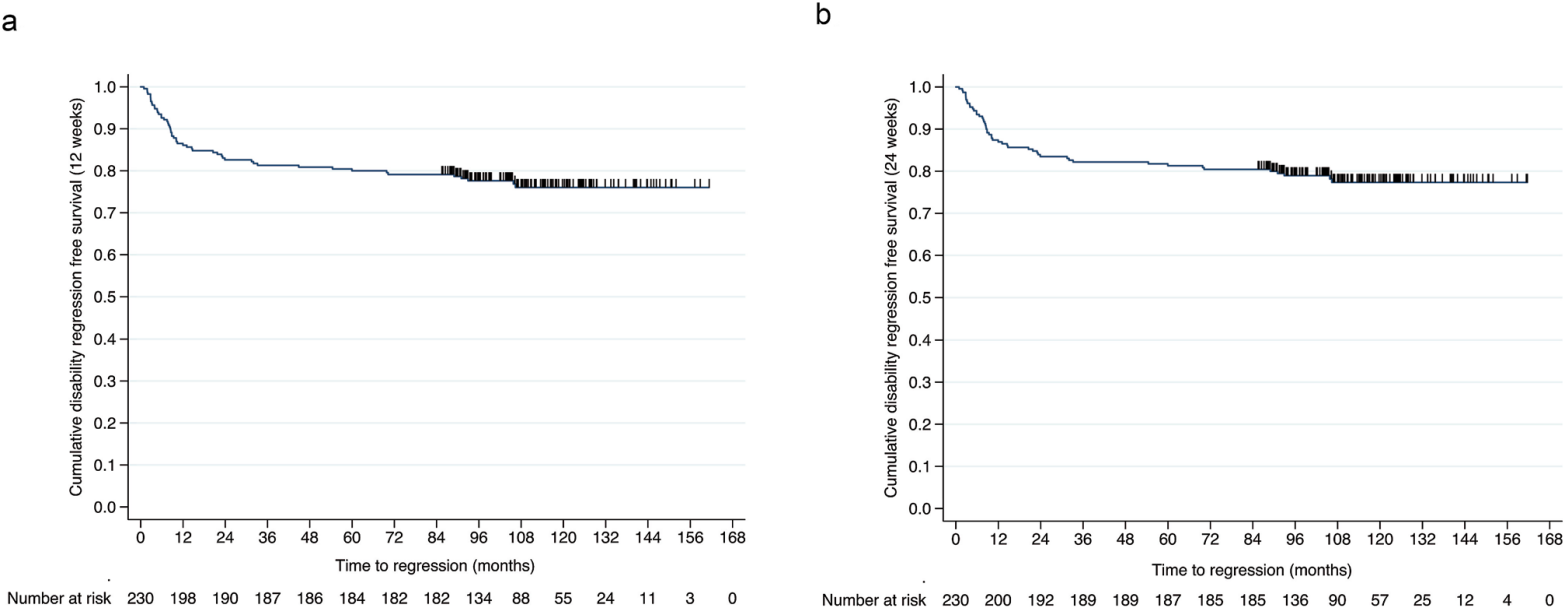
**a**, **b** Cumulative survival without disease regression sustained for 12 **a** and 24 **b** weeks during natalizumab treatment in the long-term cohort within the observation period

On the other hand, sustained EDSS regression confirmed at week 12 and week 24 was seen in 45 (23.7%) and 42 (22.1%) patients, respectively (Fig. 3a, b).

Epoch analysis regarding ARR and sustained EDSS progression and regression revealed most events during the first 2 years of NTZ treatment. 30 relapses occurred during the first, 13 during the second, 10 during the third and 23 beyond the third year. Similarly, sustained EDSS progression and regression for 12 weeks developed in 6, 12, 3 and 21 cases and 32, 8, 3 and 10 cases, respectively.

With regard to relapse activity, the baseline parameters ≥ 1 Gd-enhancing lesion(s) [incidence rate ratio (IRR) of 0.409 (95% CI 0.283–0.593), *p* < 0.001], ARR ≤ 1 in the prior 12 month before treatment initiation with NTZ [IRR of 0.353 (95% CI 0.200–0.623), *p* < 0.001] and EDSS ≤ 1 [IRR of 0.081 (95% CI 0.011–0.581), *p* = 0.012) predicted a significantly reduced relapse risk; whereas, disease duration ≤ 5 years significantly increased relapse risk [IRR of 1.851 (95% CI 1.249–2.743), *p* = 0.002] (Table 2). Analysing the total cohort, we could confirm these results and in addition, baseline parameters gender (male) [IRR of 0.751 (95% CI 0.636–0.888), *p* = 0.001] and no pre-treatment [IRR of 0.711 (95% CI 0.558–0.904), *p* = 0.006] predicted a significantly reduced relapse risk, whereas age ≤ 35 years significantly increased relapse risk [IRR of 1.214 (95% CI 1.046–1.408), *p* = 0.011] (Table 3).

**Table 2:** Incidence rate ratio (IRR) of baseline parameters predicting the endpoint relapse rate in the long-term cohort

	IRR	95% Wald confidence interval—lower	95% Wald confidence interval—upper	Statistical significance (*p* value)
Gender (male)	0.799	0.518	1.233	0.311
Age (≤ 35 years)	0.782	0.526	1.165	0.227
Duration of MS at baseline (≤ 5 years)	1.851	1.249	2.743	**0.002**
EDSS at baseline ≤ 1	0.081	0.011	0.581	**0.012**
Relapse rate ≤ 1 within 12 months prior treatment start	0.353	0.200	0.623	**< 0.001**
≥ 9 T2 lesions	1.430	0.498	4.106	0.506
≥ 1 Gd-enhancing T1 lesion	0.409	0.283	0.593	**< 0.001**
No pre-treatment	0.770	0.371	1.601	0.484

*p* value < 0.05 indicated in bold as statistical significance level

*EDSS* Expanded Disability Status Scale, *IRR* incidence rate ratio, *Gd* gadolinium, *MS* multiple sclerosis

**Table 3:** Incidence rate ratio (IRR) of baseline parameters predicting the endpoint relapse rate in the total cohort

	IRR	95% Wald confidence interval—lower	95% Wald confidence interval—upper	Statistical significance (*p* value)
Gender (male)	0.751	0.636	0.888	**0.001**
Age (≤ 35 years)	1.214	1.046	1.408	**0.011**
Duration of MS at baseline (≤ 5 years)	0.947	0.818	1.097	0.467
EDSS at baseline ≤ 1	0.412	0.313	0.543	**< 0.001**
Relapse rate ≤ 1 within 12 months prior treatment start	0.580	0.486	0.691	**< 0.001**
≥ 9 T2 lesions	1.089	0.793	1.497	0.598
≥ 1 Gd-enhancing T1 lesion	0.824	0.712	0.952	**0.009**
No pre-treatment	0.711	0.558	0.904	**0.006**

*p* value < 0.05 indicated in bold as statistical significance level

*EDSS* Expanded Disability Status Scale, *IRR* incidence rate ratio, *Gd* gadolinium, *MS* multiple sclerosis

According to these results, ARR > 1 at baseline [Hazard Ratio (HR) of 2.392 (95% CI 1.145–5.001), *p* = 0.02] and EDSS > 1 at baseline [HR of 7.666 (95% CI 1.047–56.109), *p* = 0.045] shortened and ≥ 1 Gd-enhancing lesion significantly prolonged time to first relapse [HR of 0.578 (95% CI 0.336–0.994), *p* = 0.047].

The only predictive baseline parameter for EDSS progression confirmed at 12 and 24 weeks was age older than 35 years [HR of 2.482 (95% CI 1.110–5.549), *p* = 0.027 and HR of 2.492 (95% CI 1.039–5.978), *p* = 0.041, respectively].

In contrast, no significant baseline parameters predicting EDSS regression sustained for 12 and 24 weeks were found.

The number of patients in the short-term cohort interrupting treatment was 960 (67%). Reasons for permanent treatment interruption were mainly anti-JCV antibody positivity (*n* = 445, 46.4%), the patients’ decision (*n* = 317, 32.2%), disease progression (clinical and/or MRI activity; *n* = 189, 19.7%), adverse events (AEs) (*n* = 95, 9.9%) and a stable disease (defined at the physician’s and patient’s discretion) (*n* = 35, 3.64%). Pregnancy or the wish to conceive were documented in 66 patients. The majority (*n* = 565) switched to another treatment within the AMSTR—474 (83.9%) to fingolimod, 34 (6%) to dimetylfumarate, 19 (3.4%) to ocrelizumab, 18 (3.2%) to teriflunomide, 13 (2.3%) to alemtuzumab, 5 (0.9%) to cladribine and 2 (0.4%) to daclizumab. In four patients, documentation was interrupted due to death [related (e.g., PML) or unrelated]. Treating neurologists were allowed to name several reasons per patient.

Only 74 patients (32.2%) in the long-term cohort interrupted treatment at the last follow-up. Reasons for permanent treatment interruption were mainly anti-JCV antibody positivity (*n* = 49, 66.2%) the patients’ decision (*n* = 33, 44.6%), disease progression (clinical and/or radiological activity; *n* = 19, 25.7%), stable disease (*n* = 8, 10.8%), adverse events (AEs) (*n* = 6, 8.1%) and pregnancy or the wish to conceive (*n* = 2, 2.7%).

Eleven patients switched to fingolimod, seven to dimetylfumarate, five to ocrelizumab, three to teriflunomide and one to cladribine. No patient died in the long-term cohort.

The most frequently reported adverse events (AEs) with NTZ in the short- and long-term cohorts were infections (*n* = 62/10), infusion related reactions (*n* = 54/5) and neurological disorders (*n* = 46/7). 14 cases of PML occurred so far among Austrian patients treated with NTZ.

## Discussion

In this observational study, we prospectively collected data to analyse the effectiveness of NTZ in 230 patients, who had continuously received treatment for at least 7 years, and in a second step to define baseline parameters allowing predicting a favourable or unfavourable outcome with NTZ treatment before initiation of such treatment.

Baseline parameters of our cohort were comparable to previous observational studies (Table 1) [3, 4, 7, 9, 14, 19, 20]. The ARR during NTZ treatment over the whole observation period was low, which is in line with previous studies showing a mean ARR between 0.15 and 0.3 [3, 4, 14]. Former studies found disability progression during NTZ treatment in 10–43.7% after observation periods between 44 weeks and 10 years [3, 4, 7, 19]. Sustained EDSS progression for 12 weeks was observed in 19% and for 24 weeks in 16.3% of our patients, in general yielding a low EDSS progression rate. Similar results were seen analysing EDSS regression confirmed at 12 and 24 weeks, which were 23.7 and 22.1%, respectively. This is also in line with previous studies [3, 4, 7, 9, 10, 14, 19].

In our cohort, the only baseline parameter predicting an increased risk of relapses was a disease duration of ≤ 5 years. Evidence of Gd-enhancing lesions, EDSS ≤ 1 and an ARR ≤ 1 at baseline predicted a reduced risk of relapses. Similar results were found in various other studies [3, 4, 12]. According to these results, ARR > 1 at baseline and EDSS > 1 at baseline shortened and ≥ 1 Gd-enhancing lesions significantly prolonged time to first relapse. This is in line with the previous-mentioned studies regarding the endpoint relapse activity [3, 4, 12].

In contrast, Dekker et al. in 135 patients and Sargento-Freitas et al. in 48 patients showed a favourable treatment response with higher baseline relapse rates [7, 13].

There is some discrepancy concerning disease activity as a predictor for experiencing a relapse or time to first relapse. Whereas lower ARR predicted a reduced risk of relapses and prolonged time to first relapse in our cohort, ≥ 1 Gd-enhancing lesions revealed contrary effects. Similar results were found by Bigaut et al. in the TYSTEN cohort showing a higher baseline relapse rate as a risk factor and more Gd-enhancing lesions on baseline MRI as a protective factor experiencing disease activity or progression [20]. Belachew et al. showed that patients with ≥ 1 Gd-enhancing lesions on baseline MRI tended to develop more confirmed EDSS improvement [14]. Furthermore Conway et al. revealed that Gd-enhancing lesions at baseline predicted a longer NTZ treatment, maybe reflecting a better treatment response [21].

The only predictive baseline factor for developing sustained EDSS progression was age beyond 35 years. This was in line with previous studies [11, 13]. Further factors predicting disease progression in the literature were a longer disease duration and a lower pre-treatment relapse rate [7, 8].

The reasons for interrupting NTZ in our cohort were mainly anti-JCV antibody positivity, the patient’s wish, disease progression and AEs. Subsequently 565 patients switched to another treatment. This also is in line with previous studies [21–23]. The most frequently reported AEs with NTZ in the short- and long-term cohorts were infections, which was also described in the TYGRIS study [23].

There are also limitations to our study. One limitation concerns the missing MRI data during the observational period. MRI results were only available at baseline before starting treatment with NTZ, because this information is also required for reimbursement purposes in Austria. Another aspect refers to the fact that we did not exclude the active inflammatory period at the beginning of the NTZ treatment to measure disability changes. The long observation period over 7 years should balance this possible bias.

Trojano et al. observed a significantly higher risk of 24- and 48 weeks confirmed EDSS progression in patients with on treatment relapses than in those without relapses in a cohort of 4161 patients-receiving NTZ for ≥ 24 months [10]. We did not analyse relapses during NTZ treatment in our cohort as a predictive parameter for disease progression, because our primary goal was to define predictors at initiation of NTZ treatment.

The ultimate sample size was relatively small in comparison with previous studies, but on the other hand those larger study populations are limited by shorter observation periods [3, 11, 19, 20]. Prosperini et al. also observed patients up to 7 years after starting NTZ, but here only 58 patients were still on treatment at this timepoint [15]. In the Tysabri Observational Programme (TOP) 491 patients were observed more than 8 years concerning long-term safety and effectiveness, but without defining baseline predictors [4].

Finally, our long-term cohort is a pre-selected subgroup and a bias concerning effectiveness because of the treatment interruptions before year 7 of patients with high disease activity cannot be excluded. However, we do not conclude a relevant bias regarding our long-term cohort as determined study population for the predictor analysis for several reasons.

We were able to show that the long-term cohort was rather similar to the short-term cohort (Table 1). There were four significant differences between the groups. Patients with a treatment duration ≥ 7 years were older, showed a higher baseline EDSS and experienced a higher ARR prior NTZ start. Furthermore, patients in the long-term cohort initiated NTZ more likely because of treatment failure despite prior platform medication.

ARR was 0.07 over 9.3 years in the long-term cohort and 0.2 over 3.9 years in the total cohort. Also, in the TOP study ARR did not differ significantly between the ≥ 8 years cohort and the overall population [4].

Moreover, the majority of patients interrupted NTZ due to PML-risk because of JCV-seropositivity and treatment duration of NTZ longer than 2 years. Only 19.7% stopped NTZ treatment because of disease progression, especially 4.7% due to relapse activity.

Complementary Wiendl et al. and Trojano et al. also determined a minimum time on treatment with NTZ of 2 years in their studies [10, 19].

Finally, analysing the total cohort we could confirm the baseline parameters predicting a significantly reduced or increased relapse risk.

In conclusion, our results showed a stable disease course regarding relapse activity and disease progression under NTZ treatment for more than 7 years. The main predictors for disease activity were higher relapse activity before baseline, disability measured by EDSS, shorter disease duration and the absence of Gadolinium-enhancing MRI lesions at baseline. An older age at NTZ start was the only significant risk factor for disease progression in the long-term evaluation.
